# Challenges in municipality healthcare services—The nurse leaders' perspective

**DOI:** 10.1002/nop2.270

**Published:** 2019-03-19

**Authors:** Hilde Søreide, Dagrun Kyrkjebø, Maj‐Britt Råholm

**Affiliations:** ^1^ Faculty of Health and Social Sciences Western Norway University of Applied Sciences Forde Norway

**Keywords:** management, nursing home care, nursing homes

## Abstract

**Aim:**

To examine perceptions of key challenges that nursing leadership face when organizing healthcare services in the municipality.

**Design:**

A qualitative study involving community nurse leaders (*N* = 9) in two focus group interviews.

**Methods:**

The material has been processed and interpreted in accordance with the phenomenological‐hermeneutical tradition, and this process was inspired by Graneheim and Lundman.

**Results:**

Three themes were identified in this study: (a) Tension between organizing the daily work and future challenges; (b) Challenges with recruiting enough registered nurse (RNs) in municipal healthcare services; and (c) Competence development plan—a strategic tool for nursing leadership. The municipal healthcare services need a better knowledge base with better knowledge of both the content and quality of services, organization, leadership and management, thus improving new forms of work and professional approaches.

## 1 INTRODUCTION

The organizing, managing and monitoring of healthcare services for older people has undergone significant changes in Scandinavia. These changes are based on the market‐oriented principles inspired by ideas of New Public Management (Christensen, 2013). This has resulted in different expectations of the role, skills and knowledge of leadership and management highlighting the most important challenges that leaders are facing in the nursing community (Duffield et al., 2001; Haycock‐Stuart & Kean, 2012; Holm & Severinsson, 2014). In Norway, the Coordination Reform was implemented in 2012 (Ministry of Health and Care Services, 2009 White Paper, 47), 2008–2009) and many nursing tasks have been transferred from the specialist health services to the municipal health care (Gautun & Syse, 2013). The municipal care services have become large and complex operations over the years. In order to safeguard the statutory responsibility and requirements for various quality systems such as internal control, continuous quality and patient safety work, innovative leaders on all levels are required. According to Nelsey and Brownie (2012), effective leadership is central when improving nurse retention rates and reducing the turnover of nursing staff. Good leadership plays a key role in obtaining successful change and a positive care culture (Laschinger, Purdy, & Almost, 2007) in reorganizing the work (Rokstad, Vatne, Engedal, & Selbæk, 2015) and in allocating appropriate workloads to nurses (Duffield, Roche, O'Brien‐Pallas, Catling‐Paull, & King, 2008).

## 2 LEADERSHIP AND COMPETENCE DEVELOPMENT STRATEGIES

Nursing and healthcare services have grown significantly over the past 20 years. Number of users of institution, housing, home care and practical assistance have increased from 186.000 to nearly 223,000, or by 20% in Norway (Otnes, 2015). The predictions point to varying shortages of staff in the coming years in the Nordic countries. However, the central political actors, that is governments and (professional) organizations, are especially preoccupied with the competency of the healthcare professionals (Nordic Council of Ministers, 2014). A current acute concern in Norway is the future recruitment of competent staff in this sector, particularly with regard to nursing homes, where the patients are becoming increasingly sick and frail (Dellefield, 2000; Jacobsen & Mekki, 2012; Rokstad & Romøren, 2008). Around 25% of the person‐years in care services are performed by employees without professional education in healthcare services. A concentrated effort must be made to strengthen and develop the leadership role for managers in the administration as well as in the professional municipal healthcare services due to rising expectations of high‐quality services and new tasks (p. 24).

## 3 LEADERSHIP AND QUALITY OF CARE

Ensuring quality of care for residents in the community sector is the subject of ongoing international debates (Spilsbury, Hewitt, Stirk, & Bowman, 2011). Nursing leaders occupy a crucial position in quality work in the community sector (Castle & Decker, 2011; Kjøs, Botten, Gjevjon, & Romoren, 2010). Care of elderly people is one of the national priorities, and concern has been expressed. Several policy documents in Norwegian health care emphasize quality standards for municipal care. Regulations concerning the quality of care in health and social services were issued in 1997 and revised in 2003 (Kjøs et al., 2010; Norwegian Ministry of Health & Care Services, 2003). Andersson, Frank, Willman, Sandman, and Hansebo (2018) maintained that the lack of competence was one of the most common factors contributing to serious adverse events. It seems that the challenge lies both in creating an understanding of the duty to implement internal control and to fulfil this obligation, which applies at all levels, from the municipal central management to where the patient receives the service (The Office of the Auditor General, 2016). As of January 2011, a new regulation was implemented “The guarantee for dignity” (Ministry of Health and Care Services, 2010). The purpose of the regulation is to ensure that the care for elderly persons, whether this is home‐ or institution‐based care, is organized in a way that contributes to dignified, secure and meaningful ageing. According to Haycock‐Stuart and Kean (2012), leadership and the provision of high‐quality care are important elements of NHS policies aimed at changing how municipal nursing care is delivered. The Scottish Government (SG 2010) also underlines this. The current quality agenda both in Norway and The UK are describing future directions for implementing this policy.

## 4 AIM

The aim of this study was to examine perceptions of key challenges that nursing leadership face when organizing healthcare services in the municipality.

## 5 RESEARCH DESIGN

### 5.1 Method

A qualitative enquiring research approach was selected for the study, and focus group interviews were conducted. The qualitative method is a flexible research method which unfolds throughout the research process and can thus present diversity and nuances in the material (Polit & Beck, 2017). Focus group interviews were selected in order to get collective views and the meanings that lie behind those views among the informants. Focus group interviews in this study were useful in generating a rich understanding of the leaders’ experiences and beliefs (Morgan, 1998).

### 5.2 Setting and sample

According to Krueger (1994), willingness to take part in group discussions is paramount in generating important data and using a homogenous group is crucial. The study was implemented in the municipalities of Western Norway with the number of residents being <15,000. The head nurses of the ten municipalities were contacted, and permission for the study was granted after which the informants were asked by the head nurses to participate in the study. Nine nurse leaders were included, one man and eight women, all had a bachelor degree in nursing. Four participated in focus group 1, and five participated in focus group 2. Due to illness, these numbers changed at the time of the interviews so that each group consisted of three and, respectively, four informants. Five of the nurse leaders were working in a nursing home and two within the home health care. The average age of the informants was 46.6 years with a median age of 48 years. Most of the nurse leaders had completed postgraduate education and training with a minimum of 45 credits in addition to the basic education. Table 1 shows an overall view of the informants in the study.

**Table 1:** Presentation of the informants: gender, age, post qualification (after completion of a bachelor degree) and current working place

Gender	Age	Credits	Working place
F	48	90	Nursing home
F	50	105	Nursing home
F	41	60	Home health care
M	50		Home health care
F	43	>45	Nursing home
F	33	90	Nursing home
F	61	90	Nursing home

### 5.3 Data collection

The focus group interviews were completed in the municipalities’ own meeting rooms so that the informants would not have to travel far in order to participate. Some of the participants were interviewed in their own work place. The length of the interviews was maximum 1, 5 hr. The first author of the article acted as the moderator during the interview, whereas the second author acted as co‐moderator. The moderator's role was to present the interview themes and to ensure that the participants would be able to freely discuss the different topics presented in the interview guide (Krueger & Casey, 2009; Polit & Beck, 2017). An interview guide with open‐ended questions was used in order to show the purpose of the study and the research questions. Digital sound recordings of the interviews were also made, and after the material was transcribed, it was sent to the informants for reading. No comments on the transcribed material were made by the informants.

### 5.4 Data analysis

The material has been processed and interpreted in accordance with the phenomenological‐hermeneutical tradition (Polit & Beck, 2017), and this process was inspired by Graneheim and Lundman (2004). The moderator and co‐moderator began analysing the material immediately after the interviews had been conducted. The material was transcribed by the first author and then compared to the sound recording. All the co‐authors reviewed the material from the two interviews, and the data were organized thematically according to the aim of the study. Every quote was condensed, and a code reflecting the content was given to the quotes. When the codes indicated that the content discussed the same issues, the quotes were re‐entered to have a common theme. Finally, three different themes were identified from the material: organization, recruitment and competence development (Table 2).

**Table 2:** Description of the analysis process (Graneheim & Lundman, 2004)

Meaning unit	Condensed meaning unit	Code	Category	Theme
“Although we do not have a concrete plan of competence to guide us, I still think that we work more or less consciously everyday based on our own unwritten plans.”	Despite no plan of competence, we still work more or less consciously/unconsciously based on our own unwritten plans.	Work consciously/unconsciously everyday with unwritten plans.	Leader responsibility	Organization

### 5.5 Ethical considerations

This study is part of the NursComp project and was approved by the Data Privacy Officer, at the Norwegian Centre for Research Data (project number 47191). The storage of data and the conduction of the study was completed in accordance with ethical guidelines and Declaration of Helsinki (2013).

The informants received both oral and written information about the study, and everyone signed a personal form of informed content. In the text, the data are presented in anonymous form and the informants cannot be identified in the material.

## 6 RESULTS

The results section is based on nurse leaders’ descriptions of the challenges they encounter when healthcare services are organized in the municipality. Three themes were identified through the interviews: organization, recruitment and competence development (Table 3).

**Table 3:** Summary of the results

Key points The key challenges that nursing leadership face when organizing healthcare services in the municipality are:
Tension between organizing the daily work and future challenges
Challenges with recruiting enough RNs in municipal healthcare services
Competence development plan—a strategic tool for nursing leadership

### 6.1 Tension between organizing the daily work and future challenges

The nurse leaders specified that organization was something they constantly thought of in relation to the daily operations but especially with regard to the future. One challenge regards the capacity to provide the same quality of care to more patients with the existing resources, perhaps fewer resources. It was important to be available in the ward, but they had the responsibility of organization. The informants meant that delegating tasks is one of their responsibility and thus easier to accept by the staff. The reward for this was that the nurses felt safer to delegate and transfer their tasks to others, thereby enabling them to concentrate on their own tasks.

I believe that we must work much more with this in the future and in any case focus as much on it as we currently do. Because we see that we need to use our competence in another way in order to ensure that the user, that is to ensure that the right kind of competence is used in the right place.[…………] And then one must think differently to ensure that the competence is found where the competence is necessary

The nurse leaders thought that the overall competence of the staff was good but it was difficult to recruit new personnel who possessed the required competence. All informants agreed that they needed to make changes and also change their way of management. The nurse leaders realized the importance of conveying to the consumers of the healthcare services that these cannot be passive “consumers” when they receive services from the municipality. In the future, it will be important that those who are appointed to a position (leader) are certain of what they may demand from the service consumers in order to be able to provide adequate health care to all those who need it, and on time. The informants thought that this was an important area to focus on as the right changes made might enable more consumers to manage without help for longer. The nurse leaders thought that cooperation between the different wards within the municipality was not a solution for the future but were aware of challenges that could lead to services not being provided, that is during absences due to sick leave.

[…] in the future, I think we may have to figure out how to use our competence across departments […]

An example of this was the assumption that “fewer” nurses would be responsible for more patients in the future. Their concerns were that individual nurses were given much responsibility, and this could affect the recruitment of new nurses negatively. A cooperation plan was requested by the ward managers. One of the informants had tested a system which used racks on wheels for transport between the wards. The challenge which appeared was that several wards needed the rolling rack at the same time. All the informants felt that they needed a plan for organizing the services and that this plan should come from a higher level.

### 6.2 Challenges with recruiting enough RNs in municipal healthcare services

The nurse leaders highlighted the fact that it was demanding to hire new nurses. The supply of nurses from the local area was very limited, especially in small municipalities. Vacancies were advertised but the response rate was low.

There is no point in handing out shorter shifts because it leads to displeasure among the nurses. Offering part‐time employment is also not successful considering how it is viewed as unattractive.

Wards that were undergoing a development process and constant change received more applications than other wards in the same municipality.

The informants conveyed that they had to hire more staff members based on the workload but did not have enough positions to advertise. In order to keep the nurses remaining in their positions, the leaders thought that they needed to get better at giving more challenging tasks to those recruits who wished for this. One informant thought that they were fortunate to have the competence they had in the municipality as it was important to arrange for the nurses to use their acquired competence.

Often, the nurse leaders noted the importance of being present in the ward showing that they valued that the nurses spent their time on using and improving their competence. Using the competence of the staff in the right way and to make municipal healthcare positions coveted by pinpointing the tasks to a greater extent was emphasized.

And this is related to the recruitments, it might be a bit more exciting to work as a nurse for the municipal health care services if there's more of an edge to the job

### 6.3 Competence development plan—a strategic tool for nursing leadership

Better use of the available competence in the municipalities was clearly asserted during the interviews. One of the nurse leaders mentioned that they regularly met with other colleagues and discussed issues concerning competence and competence development; they had “control” over the competence possessed by their staff. A plan for what kind of competence areas they should focus on in the future was something that needed further work. A competence plan could help the unit to become more systematic in its work and also ensure services for the consumers. Currently, the competence plan was not a tool used in the daily operations.

Although we do not have a concrete plan of competence that guides us, I still think that we work more or less consciously everyday based on our own unwritten plans.

One of the informants felt that they had a bit of a conservative work style. Another informant thought that their competence plan was practical when receiving questions about what kind of competence development the staff needed. In such cases, the competence plan was an information tool containing information about the focus areas. The ward managers lacked a plan for how to use the competence of the recruited nurses acquired. The areas of nutrition and psychiatric nursing were seen by the informants as requiring national criteria. Here they could receive support for further training according to the national Competence Lift Plan. According to the nurse leaders, acute care and palliative care were important areas to develop further and these also corresponded to the needs of the nurse leaders.

The informants conveyed that they needed to become more effective at encouraging the appointed nurses to share their competence in patient‐oriented situations and thereby lifting the competence of the whole nurse team. Informants had an on overall understanding of formal competence, whereas the informal competence, which individuals can possess, but which is undocumented, was more difficult to describe explicitly by the nurse leaders. As an example, the staff was skilful at treating wounds but had gained this skill through practical experience which was undocumented.

[…]it is to use the informal competence in a proper way, perhaps this is something one is able to contribute

In order to strengthen the healthcare services, one informant had plans on identifying the informal competence. The informants mentioned that they wished to have infinite competences; however, for recruited nurses to invest time in improving their competence they would need to be able to use their competence at work. According to the nurse leaders, the greatest need for high competence was found where the work was exigent and less attractive for the staff. One informant felt that the recruited nurses did raise their competence level but they did so in order to apply for other jobs. Therefore, there was a general uncertainty of whether a competence plan would improve the situation.

## 7 DISCUSSION

The results of this study highlight what Haycock‐Stuart and Kean (2012) also assert in their study, which is that the current service organization and delivery at the frontline often conflicts with the different policy agendas. The nurse leaders are at intersection point between having the responsibility for implementing the various strategic aims while still giving the highest priority to organizing the resources for the daily care. Nursing leaders occupy a crucial position in quality work in the community sector (Castle & Decker, 2011; Kjøs et al., 2010). The nurse leaders could benefit from guidance/consultation in daily operations in order for the organization to attain the professional goals it has set for itself (Tingvoll, Saeterstrand, & McClusky, 2016). More competence was not necessarily needed for strengthening the services according to the informants, but instead changing how the available competence was organized. Earlier studies confirm this and state that lateral task moves between professions may make the municipal healthcare services more effective and increase the availability of these (Tyrholm, Kvangarsnes, & Bergem, 2015).

The informants also specified that they lacked an organizational plan from the municipality.

Bondas (2006) and Mc Kenna, Keeney, and Bradley (2004) indicate in their research that the nurse leaders felt that they did not manage to be strong leaders and that they also had little authority. They were caught between the financial managers, laws and traditions which made the decision‐making processes difficult. Therefore, higher demands must be placed on the management in order to raise the service quality. Organizational culture and leadership are important when promoting and encouraging dialogue and creative, innovative methods to improve patient care, nurses' job satisfaction and recruitment and retention (Coventry, Maslin‐Prothero, & Smith, 2015).

Organization was particularly important with regard to the future. The informants mentioned that they had the responsibility for organizing the services but that there was little time for this during the daily operations. One area of responsibility was to work systematically with quality and patient safety. In order to have more time for professional healthcare matters, the informants found that the nurses felt safe delegating their tasks. The importance of developing a safe and trustful culture where the nurse leaders are able to influence and contribute to the empowerment of staff and organization becomes crucial (Holm & Severinsson, 2014). In addition, leaders must exercise caution when making decisions, ensuring that fairness and equitability exists among staff, and that ethical standards are upheld on a continual basis (Kane‐Urrabazo, 2006). One way of improving the professional environment is to improve the cooperation between different professional groups by having them share the location. This way related healthcare services would be coordinated better. There is a need for re‐thinking and paving the way for new roles and increased cooperation in order to reach everyone dependent on help (Tyrholm et al., 2015). Organization between the municipal departments was one future solution which was presented in this study. A connection to similar professions, work place and age are factors affecting the competence level of the services provided (Bing‐Jonsson, Hofoss, Kirkevold, Bjørk, & Foss, 2016). One challenge affecting the cooperation across the provided services is the vulnerability due to sick leave absences.

The changing landscape of municipality health care stresses the importance of efficiency, effectiveness, accessibility, increased consumer participation and choice and accountability (Dickson & Coulter Smith, 2013; Scottish Government, 2007). Risk management is also paramount in this type of service provision that sees more unqualified staff and fewer specialist practitioners delivering care and meeting the needs of vulnerable people in municipal health care (Dickson & Coulter Smith, 2013).

Advertised nurse vacancies received few applications, and the retention percentage of these positions had a great impact on the response that the informants got. To attract young applicants to the municipality is greatly a matter of how the working conditions correspond to the applicants’ expectations and also how municipal health care is presented during the nurse education (Bakkeli, Sterri, & Moland, 2016; Prentice & Black, 2007). Recruiting and retaining nurses is challenging, and it is disquieting that nurses leave their jobs in the municipalities (Cooper et al., 2017). It is likely that there is a relationship between high turnover rates and nurses' perceptions of low competency in long‐term care (Stanyon, Goldberg, Astle, Griffiths, & Gordon, 2017). However, leadership focusing on task completion alone is not sufficient to achieve optimum outcomes for the nursing workforce (Cummings et al., 2010). Developing competencies will facilitate professional development, have an impact on career pathways and increase the status of work (p. 582). The lack of competence and incomplete documentation of competence has been reported as the most frequent contributing factors to serious adverse events (Andersson et al., 2018).

Informants who were working in departments undergoing constant changes development received more applications to the advertised positions. In order to retain staff, they found that employees who wished to have more challenges should be given the opportunity to take them. Young recent graduates give importance to exciting work tasks, opportunities for self‐development and a strong professional work environment when they evaluated potential challenges in the municipality, but they have the impression that it is a smaller work community (Bakkeli et al., 2016).

In order to optimize the competence requirements, the existing literature suggests performance appraisal, training needs analysis, professional development plans, structured mentoring or coaching, identification of career paths, paid study leave and support by the leaders (Coventry et al., 2015; National Review of Nursing Education, 2002).

Recruitment is central and having both recent graduates and experienced staff is necessary in order to create high‐quality services. Research indicates that those with the longest years of service also had the lowest competence as they have not had the opportunity to keep themselves updated (Bing‐Jonsson et al., 2016). The informants sought after a stronger organizational plan for what type of key competence should be prioritized in the future. For competence development to become a natural part of the daily work, nurses need assistance from the managers in order to plan their tasks and organize the services (Cooper et al., 2017; Kyrkjebø, Søvde, & Råholm, 2017).

The informants did not use the competence plan in the everyday work. By focusing on the young and recent graduates in the competence plan, the focus in the recruitment praxis is consequently also stronger on the young (Bakkeli et al., 2016). The informants also wished for clearer communication and to get greater certainty of what the priority areas were in the municipality before engaging in competence development. In the interviews, it became clear that the appointed nurse staff must be able to share new knowledge in the wards which would strengthen the services as a whole. Collective competence is vital in order to secure the services (Bing‐Jonsson et al., 2016). Good collegial relations are fundamental in terms of keeping one's professional skills updated as well as learning practical procedures from one another and finding the time for reflection (Kyrkjebø et al., 2017). The nurse managers play an important role when planning educational programs for practicing RNs with regard to increased attendance, financial investment and the structure and content of the specific programs (Bourbonniere & Strumpf, 2008). The informants underscored that there should be a greater focus on the competence acquired through practical experience and this was undocumented.

## 8 CONCLUSIONS AND IMPLICATIONS FOR NURSING MANAGEMENT

There is much good work done in the municipalities, but there are also several challenges. To strengthen, the competence requires systematic measures that make it attractive to work in the municipalities that will increase and develop the competence of existing personnel and to use the current skills. Also on the municipal leadership level, there is a need of changes and increased competence in the future. Competence in management, planning, quality improvement work, organization of services, public health work and cooperation are currently given high priority. The municipal healthcare services need a better knowledge base with better knowledge of both the content and quality of services, organization, leadership and management thus improving new forms of work and professional approaches. The nurse leaders are at a cross point where responsibility for implementing the strategic aims while also giving the highest priority to organizing the resources for the daily care. It is important that the leaders’ voices are heard when planning future reforms which encompass competence development and leadership. The leader's important role is to support the collegial community and to promote raising the quality of the services. The responsibility lies with the leaders to market municipal healthcare services as an employer and to show that personnel with advanced competence will be needed in the future.

### 8.1 Limitations

This study was conducted in relative small municipality with few nurse leaders. The results may therefore deviate from results of similar studies conducted in larger and central municipalities. According to the small sample in this study, it is difficult to generalize the results to a larger population.

## CONFLICT OF INTEREST

The authors declare no conflict of interest.

## AUTHOR CONTRIBUTIONS

HS and DK contributed to data collection and ethical clearance for this project while all authors contributed to methodology, literature review, data analysis and writing of the final manuscript.
